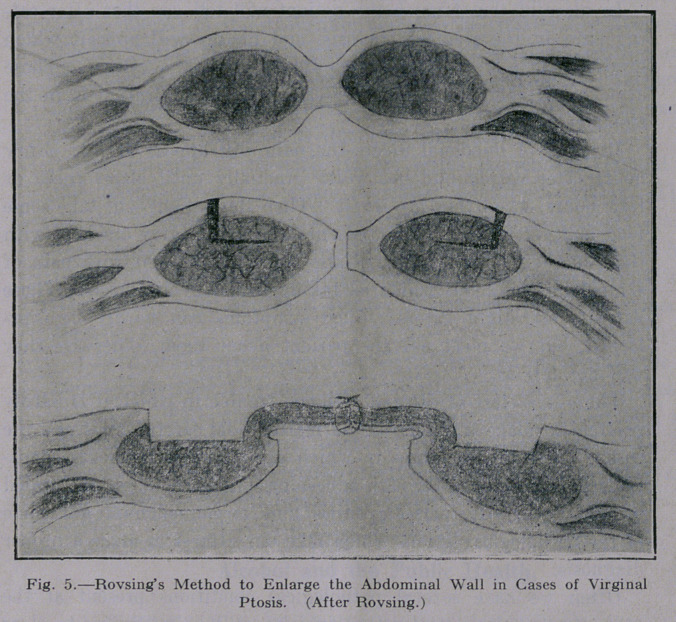# Stomach Troubles*Read at meeting of the Central Texas District Medical Society at Hillsboro, Texas, July, 1914.

**Published:** 1915-01

**Authors:** F. C. Floeckinger

**Affiliations:** Taylor, Texas


### Stomach Troubles.*

BY F. C. FLOECKINGER, M. D., TAYLOR, TEXAS.

In presenting this paper before this honorable body, I wish at first to apologize for the title I selected. It is a fact that a great many cases are coming to us with stomach trouble. If I should do justice to this paper, I would have to write a book on the subject and, therefore, I will not dwell a great deal in bibliography, but will give you some of my ideas gotten from practical experience, put them before you in a common-sense way, and let you decide as to the value of those points.

Our literature on stomach trouble is immense and the textbooks go so much into details that the general practitioner is like

*Read at meeting of the Central Texas District Medical Society at Hillsboro, Texas, July, 1914.

a ship lost at high sea. Therefore, I selected this title, which will be severely criticised as unscientific, but, nevertheless, I selected this catch title to get you to listen to it, and I will try very hard to give justice to the paper. If I don't then don't condemn me, but just consider that I was trying hard, and there is nothing like trying.

It cannot be denied that many cases of stomach troubles come to us which have made the rounds from physician to physician. Unfortunately, they often never have been benefited by any treatment, and to the primary trouble of the patient a secondary mental depression takes place, which taxes the skill and patience of the physician to the utmost.

We all know that the beginning of a tuberculosis will manifest itself very often with stomach trouble. I have observed this so often in my years of practice that I may consider this statement as a fact. Then we have Didles' crisis due to obstruction of the ureter by some cause, as the case I am presenting. You will pardon me to present this case in detail, but the history of the case and the findings are so interesting that I could not help thinking that it will be interesting to all of us to take the case up in detail.

The patient, a woman of forty-three years of age, was referred to me by a physician for treatment. The patient has been afflicted with periodical pains in the right side and stomach for twenty years. Those pains would come on suddenly and always would start in the right iliac region, extending upwards towards the umbilicus and finally ending in the epigastrium. The pain would then be most severe in the epigastric region, and then violent vomiting of bilious material would take place. As soon as vomiting started in, the patient began to feel relief from the pain. The patient states that when the vomiting starts in she will be able to feel and see a swelling in the right iliac fossa, which would reach down into the pelvis. Please observe: No pain in any of the lumbar regions, neither right nor left. The physician would give her some mild chloride, and in about twenty-four hours the tumor would disappear entirely and the patient would feel well again for a time until another spell would come on. Quality as well as quantity of food has nothing to do with the oncoming of the attack. There is no specified time before or after meals that the pain would appear. There is no tenderness in the epigastric region, no pain over the region of the gall-bladder, and percussion of the gall-bladder is not painful. Fist percussion of the kidneys is absolutely painless. The history does not give any points as to involvement of the urinary tract. There were absolutely no symptoms of frequent urination or painful urination, or pain in the bladder or urethra. On physical examination the most tender spot was over McBurney's point. No history of any appendical troubles at all. History never tells of any bleedings from the bowels shown by the passage of tarry, slimy stools, and there was never any coffee-

Fig. 1.—Calculus in Ureter.

ground vomiting. Heart, lungs, liver, spleen normal. Genital tract normal. Patient has not past the climacterium. Chemical analysis of the stomach content did not show any pathological condition of the secretion. Blood examination did not reveal anything important. No occult blood was found in the examination of the fecal matter, which was made repeatedly. The urine showed an immense amount of pus and bacteria, but no blood. Examination for tuberculosis was not made.

The woman was kept under observation for a few days at my institution and I had the opportunity to see her in one of the attacks. It came on in exactly the way described. I could observe the bulging of the right iliac region and the palpitation of the mass, which was somewhat tender, showed fluctuation. The palpating of the kidney showed a large tumor, which could be moved freely upwards and towards the middle line. It did not feel like the kidney substance, but more like a cyst. The next step was to find out if the tumor was intra- or retro-peritoneal. A large colon tube was inserted into the rectum and with a bicycle pump the large intestines were filled with air. You could observe the passing of the air in the large intestines on the abdominal wall, and finally it distended the ascending colon in front of the tumor. This proved sufficiently that we had to deal with a mass which is located retro-peritoneal. Cystoscopic examination showed a pretty normal bladder, free of any trabeculations. Ureter openings normal. The left ureteral catheter entered the pelvis of the kidney without any trouble and clear urine flowed from same; the right ureteral catheter was stopped in its forward movement about two to three inches after entering the ureteral opening. An X-ray picture was taken and a calculus was found to obstruct the passage of the ureteral catheter. (Fig. 1.) Both ureters were then injected with 15 per cent cargentos and another picture taken (Fig. 2). This second picture is very interesting. It shows at first the strictured part of the ureter, then right behind the calculus, and third it shows the immense dilatation of the ureter.

A diagnosis of impacted calculus of the mulberry type in the right ureter just over the pelvic brim and a secondary hydronephrosis of the right kidney was made. The functional test of the left kidney showed normal output of urine and normal function, but there were absolutely no functional tests obtained from the right kidney. No urine escaped from this ureter; only a little thick whitish liquid. At operation I removed an immense hydronephritic kidney and an immense dilated ureter with the calculus (Fig. 3.) The operation was a very simple one and all the stomach trouble of this woman disappeared like magic. In a personal letter the family physician wrote me that very likely I would have to deal with a case of appendicitis or gall-stones.

I think the case is worth while to present in detail, as the history and the physical examination revealed such unusual symptoms.

Tabetic crisis, pernicious anemia, pathological conditions of the central nervous system, diseases of the gall-bladder and bile ducts, pancreas, chronic appendicitis and pericolitis have all been classified as stomach troubles. Therefore, it is of the utmost importance that we eliminate all the reflex conditions which are giving us symptoms of derangements of the stomach.

Fig. 2.—Collargol sol. in dilated ureter above stone.

It is astonishing how many cases of stomach trouble are now coming to the surgeon. Every year adds more diseases of the stomach to the surgeon, and cases that have been treated in the medical wards fifteen to twenty years ago are now turned over to the surgeon and are cured; whereas before they were chronic invalids and made their rounds from hospital to hospital.

These cases are referred from the family physician to the stomach specialist, and he again refers them to a sanitarium, where the patients receive proper treatment with the aid of proper diet—hydrotherapy, massage, electricity, etc. They improve for a while and later the same symptoms complex again appear and they are invalids.

The painstaking work of Mayo-Robson, Monyhan and Patterson of England, of the Mayos and Deaver and Murphy of America, and of Rovsing of Copenhagen the last ten years has added very much to the successful treatment of these unfortunate cases.

Unfortunately there has too much stress been layed on the laboratory findings of the stomach content. In our days, where we have the fluoroscope, the radiographs in connection with the laboratory findings, the history of the case taken down and studied in detail, and the improved physical examination, we are or should be able in nearly all cases to make a proper diagnosis. But the laboratory findings must fit with the other diagnostic accessories, and the diagnostician must be able to interpret the X-ray findings, the fluoroscopic picture, and must work on it, until he is satisfied that the whole symptom complex, the laboratory findings, the fluoroscopic picture, the radiograph and the physical examination correspond. If they don't, then there is something wrong.

I can not go into details on all stomach diseases, but will select several most important types for the benefit of the general practitioner so he will be able to utilize those points in making a correct diagnosis.

Every one of us has been consulted by patients who have been making the round from one man to another, in which the diagnosis of neurasthenia has been made. I am coming to the conclusion that primary neurasthenia is a very rare disease. In nearly all those cases there is some other cause for the nervousness, but the straight statement is simply this: We have not been able to make a proper diagnosis, that's all.

Many of these cases will complain of stomach trouble, constipation, periodic vomiting, cardialgia, eructation, and, if a female irregularity of menstruation or amenorrhea. These patients are very slender, have a pinched expression, suffer from headache. At times they can eat anything without hurting them, and then again they are not able to eat anything. When we get through examining these patients we will find that they are suffering from a gastroptosis, enteroptosis and a prolapse of nearly all the intra-abdominal organs. A very simple operation will cure nearly 70

per cent of these cases. There is one place in Europe where this condition has been studied more thorough than in any other clinic in the world—the clinic of Professor Rovsing of the University of Copenhagen in Denmark. That man has devoted nearly fifteen years to the study of these cases and his method of surgery has brought such good results. Coffey of Seattle, Washington, has advocated gastropexie and colpopexia, but the percentage of cure (permanent) is very small. Stengel, Bier and Beyea have utilized the omentum minus and ligamentum gastro-hepaticum for the purpose of lifting the stomach up. According to Rovsing this method

Fig. 3.—Kidney removed. Showing rock in lower end of ureter.

is not followed by permanent results, because it will be found on exploring those tissues, that they are as thin as tissue paper and give way again. Coffey's operation, which consists of attaching the omentum majus to the anterior abdominal wall, has also not proved a success. (Fig. 4.)

Rovsing classifies these cases of ptosis into virginal and maternal. The virginal type of ptosis generally starts in women with the time of puberty. After each menstruation these patients would complain of pain, which is not located entirely to the small pelvis, but spreads out to the flanks. They suffer from cardialgia, nausea, frequent vomiting and constipation. Their condition grows worse, and finally they are not able to attend to any duties. All these patients suffer from headaches, which are due to auto-intoxication from stagnation of fecal matter in the large intestines. Indican is constantly found in the urine, and occasionally we find also acetone. Quality and quantity of food have nothing to do with the severeness of the symptoms. These women had their uterus and ovaries removed unnecessarily, because the diagnosis of infantile uterus was made.

In all these cases we find the genital organ not properly developed, but this non-development of these organs is secondary, and a removal is improper treatment. Some diagnose the case as ulcer of the stomach, duodenal catarrh, chronic nervous dyspepsia, chronic gastritis, etc. The patient is put to bed and Leube's treatment for ulcer of the stomach instituted, which consists mostly of milk and proteids. The patient does not show any improvement, and the constipation is as bad as ever, therefore the diet is changed and the patient is put on stewed fruit, prunes and Graham bread, and kept in bed. He begins to improve to a certain extent, and is finally discharged. But in a short time these symptoms return as bad as ever. When we get such a history of a patient we will be pretty sure in looking for a general ptosis, which may affect the stomach and colon alone, or also cause a hepatosis and nephroptosis with it.

Maternal ptosis does not produce such severe pains as those cases of virginal ptosis, because in a virginal ptosis there is a crowding of all the organs. In those cases it sometimes becomes necessary to enlarge the wall of the abdomen by a plastic operation. (Fig. 5.) Sometimes a resection of a pedunculated left lobe of the liver must be done to give the stomach place. In all these cases the ptosis is due to tight lacing, crowding the viscera and causing a narrowing of the lower part of the thorax and hypertrophy of the left lobe of the liver.

These cases cannot be treated in a routine manner, but each individual case must be studied thoroughly, otherwise it will be a failure. In these cases the Ewald's test breakfast will show a nearly normal acidity of the stomach content or a hypoacidity, sometimes even showing complete achylia, but never any hyperacidity, provided there is no ulcer formation combined with the ptosis. There is no occult blood found in the fecal matter or stomach content. We next give a modified Bourget's test meal to test the mobility of the stomach. This meal consists of 250 c.c.

of gruel, 75 grams of minced meat, 50 grams of bread, one-half ounce of raisins with large seeds and 10 boiled prunes. If after six hours after washing out the stomach we find no remnants of this meal in the stomach we may exclude retention. After washing out the stomach we may distend it with air and execute scraping auscultation. This auscultation is a very simple procedure and gives us a method by which we may outline the stomach. The phonendoscope is placed at the top of the cardia within the

Fig. 4.—Coffey's Operation for Gastroptosis.

curvature of the left rib while the patient is standing. The phonendoscope is pressly firmly down and with the finger nail of the other hand scraping movements are made in a radiating direction away from the phonendoscope. We will hear the difference in the sound as soon as we pass the outline of the stomach. This method is a great help to make a diagnosis, but not in all cases. If the colon is distended and lying in front of the stomach we are not able to outline the lower border of the stomach. Therefore, we come to the next diagnostic method, namely, the administration of two ounces of bithmus subcarbonate in a dish of cream of wheat. We can then study with the fluoroscope or by taking systematic X-ray pictures. We have found that the taking of a picture right after the ingestion, six hours later, and another twelve hours later, will give us a very clear idea as to the diagnosis of the case.

In all our cases of gastroptosis we have never found any stagnation of bithmus six hours after the taking of the bithmus. We must differentiate between a dilated stomach and a gastroptosis. In a case of dilated stomach we will always find a stagnation of bithmus; in a gastroptosis we will not only find the greater curvature of stomach a few inches above the os pubis, but we also find that the lesser curvature of the stomach is below the umbilicus. There is a dragging down of the whole stomach due to a laxity of the ligaments holding up the stomach. In a case of dilatation of the stomach the lesser curvature will be in the natural place.

Dilatation of the stomach may be classified in the following way: (1) Atonic dilation and (2) obstructive dilatation. The atonic form is found is cases of chronic gastritis, mostly in chronic alcoholic subjects. The obstructive type is found in cases of acute ulcer of the stomach and in malignancy. If we take an X-ray picture of the large intestines twelve hours after the ingestion of the bithmus meal we will observe their position. We may add to the clearness of the picture if we inject into the colon a mixture of bithmus subcarbonate in a suspension of mucilage of acacia, but generally it is not necessary to do this.

In cases of enteroptosis we will see that the transverse colon is suspended like a bridge at the hepatic and splenic flexures, and the middle portion is sagging down. This sagging down of the colon may be primary or secondary. If primary, it is due to the dragging down of the colon by the prolapsed stomach; if secondary, it may be due to the relaxity of the intestines. It would take a paper for itself to go into the details of the causes, and therefore I will not dwell in detail on the subject. Those X-ray plates will show us at a glance why those patients are suffering from constipation and stasis with all those reflex symptoms of auto-toxemia.

Another factor in the physical diagnosis in these cases is the pulsation of the descending aorta if the patient lies plane on the examination table. You will be able to feel the pulsation of the aorta plainly with the fingers, but as soon as you put your patient in the extreme Trendleburg's position the pulsation will disappear.

This is explained very easily. The stomach which fills the epigastric region has prolapsed, giving the aorta no organs to overlap, and, therefore, the pulsation can be felt clearly. As soon as the Trendlenburg's position is instituted the stomach will fall into the normal position and the pulsation disappears.

The pain in these cases is mostly located in the left of the epigastric region, but chronic gastritis and ulcer of the stomach have also their seat of pain in the left epigastric region. In duodenal

Fig. 5.—Rovsing's Method to Enlarge the Abdominal Wall in Cases of Virginal Ptosis. (After Rovsing.)

ulcers the pain is always located to the right of the median line about one inch above the umbilicus.

From all these observations given you come to the conclusion that a systematic examination is of the most importance to make the proper diagnosis in diseases of the stomach. It is especially the history of the case which is so essential to bring light on the subject. In ulcer of the stomach, for example, we always have a hyperacidity except in cases in which malignant degeneration has taken place. In ulcer of the stomach we find occult blood in the stools, and sometimes in the stomach content. Cachexia is present not only in malignant diseases of the stomach, but also in extreme cases of gastroptosis and ulcer of the stomach.

In cases in which malignant degeneration takes place the picture of the laboratory findings is entirely changed, the hyperacidity will disappear and we will observe a complete absence of free and hydrochloric acid. Lactic acid will be present; sarcine will be found. The Boas Oppenheimer bacillus will be found. The symptoms will keep up for a while with those of a chronic ulcer of the stomach.

In ulcer of the stomach the patient will tell you that after eating pain in the stomach, pyrosis, and nausea will appear, which gradually gets worse and is relieved by food or alkalines. Those attacks will come on right after eating if the ulceration is in the stomach.

In cases of duodenal ulcers the pain will appear at first a few hours after eating and get worse gradually until about three or four hours, when they are worst. Then the patient will take some food and get relief. Those patients will often carry crackers with them in their pocket, and will also wake up at night with pain of a gnawing character, which on taking food will disappear. Those patients are night eaters. Such symptoms can hardly be overlooked, but we must ask the patient about them, otherwise they will not tell us.

There is a class of cases of stomach trouble in which it is hardly possible to make a diagnosis, no matter how careful we study the case. These are the cases in which secondary pathological conditions have developed through adhesions, produced by an old healed ulcer. Pericolitis, pericolecystitis and megaduodenum are some of the pathological lesions which take our utmost to make a proper diagnosis.

Often we have several pathological conditions present at the same time, and an exploratory incision will be necessary to clear up the diagnosis. In all cases in which an exploratory incision is made it will be a great advantage if the surgeon applies the method of Rovsing in directly inspecting the inside of the stomach by making an incision in the stomach large enough to insert a number 26 American Brandsfords Lewis cystoscope through the opening, inflate the stomach with air and illumine the whole inside of the stomach and duodenum. By this we will be able to localize pathological conditions of the inside of the stomach and duodenum which could not be diagnosed by simple inspection and palpitation. The incision does not increase the danger of the operation and will be worth trying in each case in which the slightest doubt of diagnosis exists. Small ulcers in the ridges of a prolapsed stomach will be observed and can be excised in addition to performing gastropexie.

We have a type of duodenal ulcers which cannot be diagnosed microscopical, and they also do not give the typical picture of a duodenal ulcer; and if they do, on exploration they cannot be found. These cases are often diagnosed as duodenal catarrh, but I think that the late investigations made on this subject by Patterson and Eiselberg, of Vienna, will show post-mortem a necrosis of the mucosa of the duodenum and may be classified as duodenal ulcers. These cases will give symptoms of vomiting of bile and pain just as an ulcer, but even with the help of Einhorn's duodenal cup and test no occult blood can be demonstrated. These cases will be greatly benefited by duodenal alimentation.

Another type of stomach trouble which is frequently found is the nervous dyspepsia, where increased mobility is found. This can be diagnosed plainly by the bithmus meal and X-ray plate. In these cases we find that in three to six hours all the bithmus has passed the ileo-cecal valve and can be found in the large intestine. These cases will give us a picture of hypoacidity of the stomach content.

I have in this paper only referred to the most important type of stomach troubles, and must close my paper, which is by no means complete. The points I have given you are mostly of my own experience, and I thought it worth while to present them to you for discussion so that the general practitioner may profit by same and will be able to benefit those unfortunate sufferers.

## Figures and Tables

**Fig. 1. f1:**
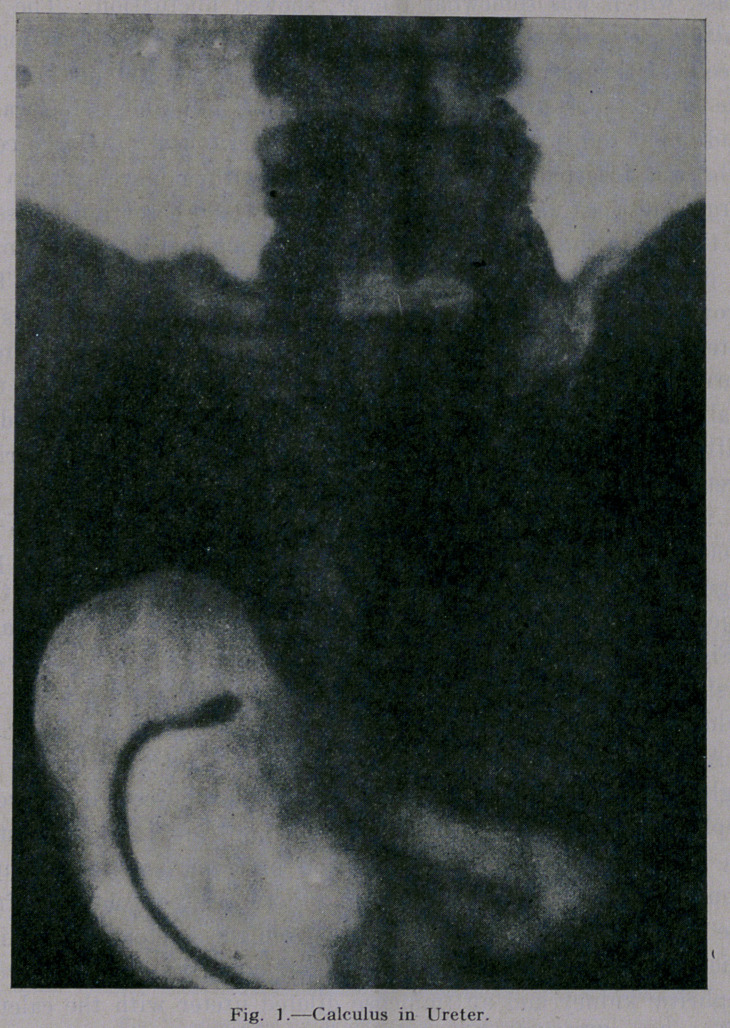


**Fig. 2. f2:**
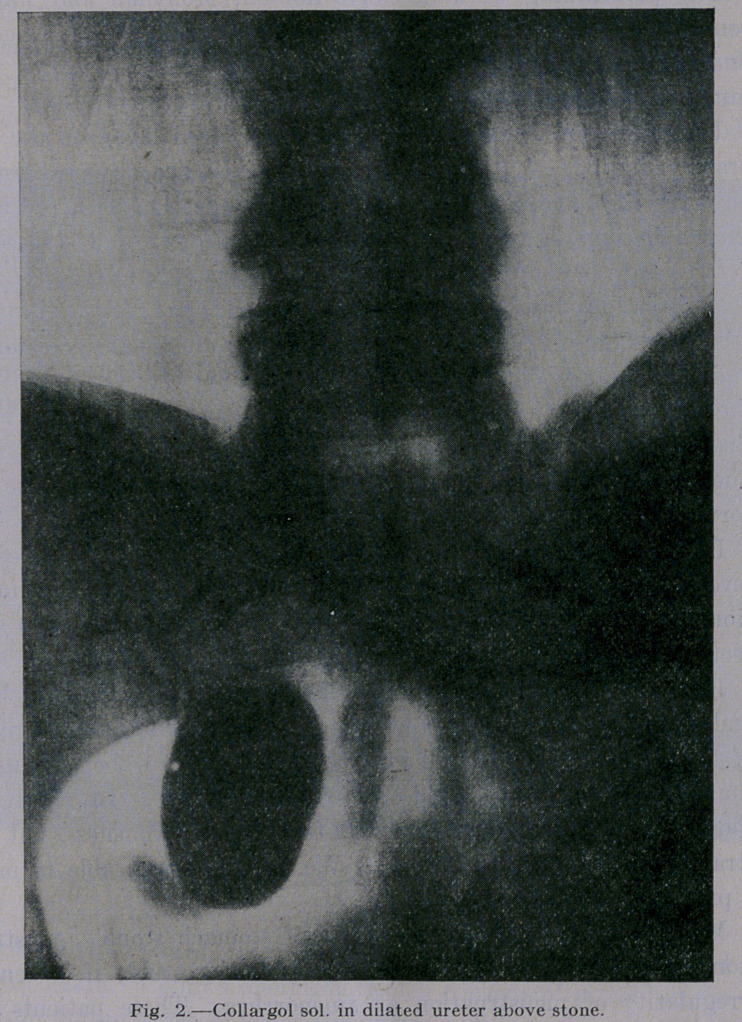


**Fig. 3. f3:**
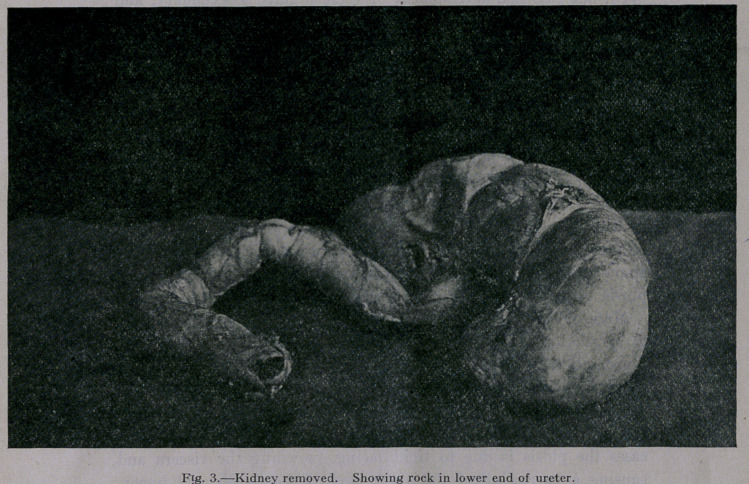


**Fig. 4. f4:**
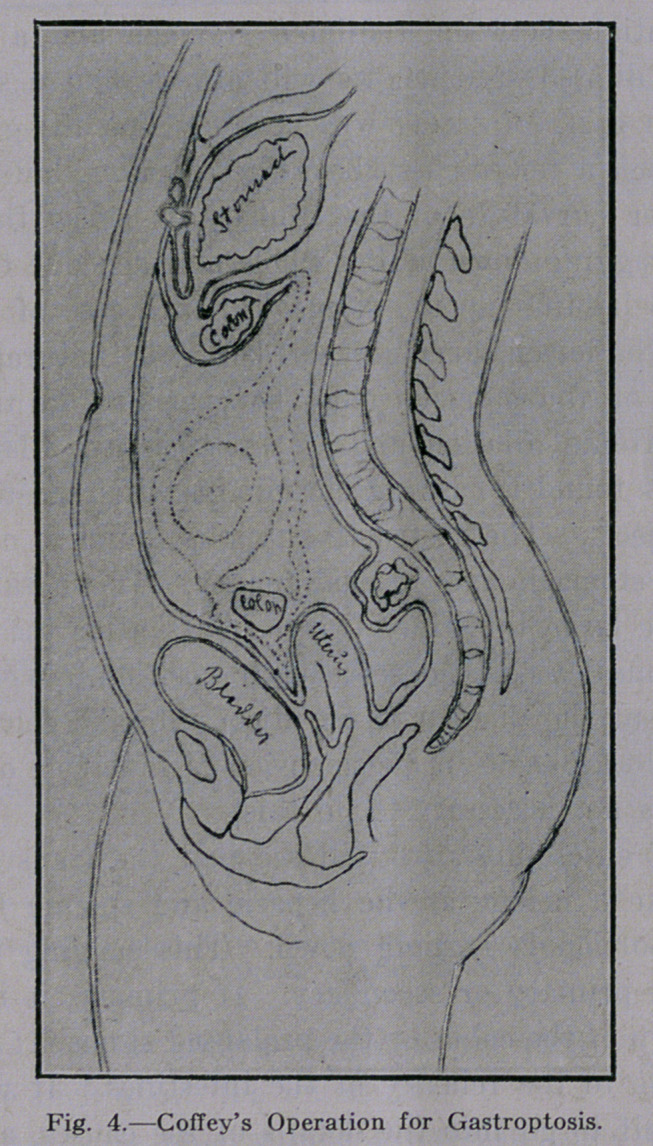


**Fig. 5. f5:**